# Post-Paralytic Knee Arthropathy Following Spinal Ependymoma Resection Treated With Bilateral Hinged Total Knee Arthroplasty

**DOI:** 10.1016/j.artd.2026.102058

**Published:** 2026-06-02

**Authors:** Jessica Nelson, Austin Post, Michael Miller

**Affiliations:** Department of Orthopaedic Surgery, University of Arizona-Tucson, Banner University, Tucson, AZ, USA

**Keywords:** Hinged total knee arthroplasty, Complex primary knee arthroplasty, Post-paralytic knee arthropathy, Neuroarthropathy

## Abstract

Post-paralytic knee arthropathy (PPKA) is a rare and underrecognized cause of progressive joint degeneration, driven by chronic muscle imbalance that leads to abnormal joint mechanics and loading patterns. We report the first known case of bilateral PPKA following spinal ependymoma resection treated with staged hinged total knee arthroplasty (H-TKA). The patient developed severe, progressive deformities over 18 years with preserved sensation, distinguishing this condition from Charcot arthropathy, which is characterized by rapid joint destruction and profound sensory loss. Staged H-TKA restored alignment, improved stability, relieved pain, and enabled functional recovery. This case highlights the unique modern pathophysiology of PPKA and supports H-TKA as a viable treatment option.

## Introduction

Hinged total knee arthroplasty (H-TKA) represents the highest level of constraint in TKA and is reserved for complex knee reconstructions. In primary TKA, hinged implants may be indicated in patients with neuromuscular instability and severe deformity, such as those with substantial sagittal and coronal plane deformity and collateral ligament deficiency. Post-paralytic knee arthropathy (PPKA) is characterized by slowly progressive deformity due to motor imbalance with preserved sensation, in contrast to Charcot arthropathy, which features rapid joint destruction secondary to sensory loss. PPKA can occur secondary to spinal cord pathology or peripheral nerve injury and leads to abnormal joint loading and mechanics over time due to muscle imbalance.

PPKA of the knee secondary to spinal cord tumor resection has not been previously described. We present a unique case of bilateral end-stage knee osteoarthritis in the setting of a spinal ependymoma, characterized by markedly asymmetric deformities requiring staged bilateral H-TKA. The patient had passively correctable but severe deformities, including 50 degrees of varus collapse with 20 degrees of recurvatum in the right knee and 50 degrees of recurvatum in the left knee. This case highlights the distinct pathophysiology of PPKA compared with Charcot arthropathy and supports H-TKA as the most appropriate reconstructive option in appropriately selected patients.

## Case history

A 72-year-old female with a history of hypertension, hypothyroidism, hyperlipidemia, and obesity (body mass index, 34) underwent resection of a malignant spinal ependymoma in 2007 (Fig. 1), after which she developed chronic asymmetric lower-extremity weakness most pronounced in the quadriceps and progressive bilateral knee deformities and pain, resulting in functional decline and eventual loss of ambulation. She became walker dependent shortly after tumor resection and remained so for approximately 13 years (2007–2020) before progressing to motorized wheelchair dependence by 2020. Figure 1 demonstrates the spinal ependymoma at initial diagnosis, while subsequent imaging included in this image series shows sustained remission.

**Figure 1:**
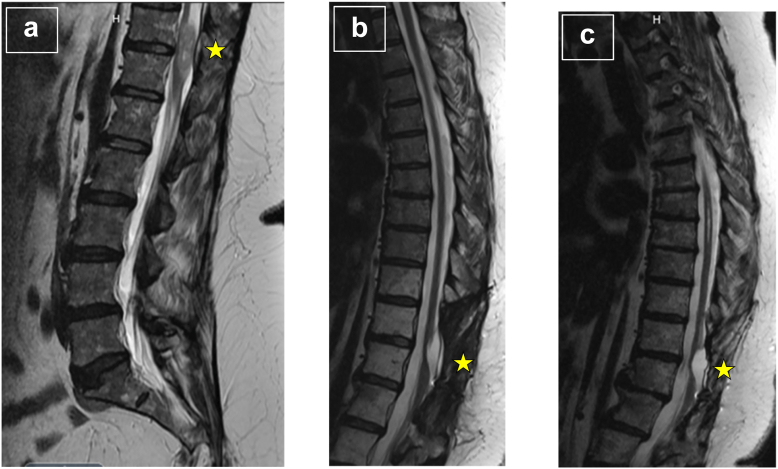
Sagittal T2-weighted magnetic resonance imaging of the thoracolumbar spine demonstrating spinal ependymoma at initial diagnosis and long-term postoperative stability. (a) Preoperative magnetic resonance imaging obtained in June 2007 showing a spinal ependymoma centered at T11–12. (b) Postoperative MRI from 2010 and (c) surveillance MRI from 2024 demonstrating stable postoperative changes without evidence of tumor recurrence. A persistent dorsal intradural loculation at T11–12 with adjacent spinal cord thinning and contour deformity has remained unchanged for more than 10 years following index resection.

Because of the complexity of her condition, she underwent multiple unsuccessful consultations with community orthopaedic surgeons, and the patient was ultimately referred to our tertiary care academic institution in 2025. The patient was previously fitted with a custom thigh-knee-ankle-foot orthosis. This was too cumbersome to use and did not provide appropriate stability for ambulation. She was told in previous conversations that there was no surgical intervention available. She also received multiple corticosteroid injections for pain. Radiographs available in the medical record before her presentation included bilateral knee imaging from 2017 and 2020. Radiographs from 2017 were grossly normal (Fig. 2). Weight-bearing radiographs in 2020, which coincided with the patient’s progression to wheelchair dependence, demonstrated varus collapse bilaterally in addition to right medial tibial metaphyseal bone loss (Fig. 3).

**Figure 2:**
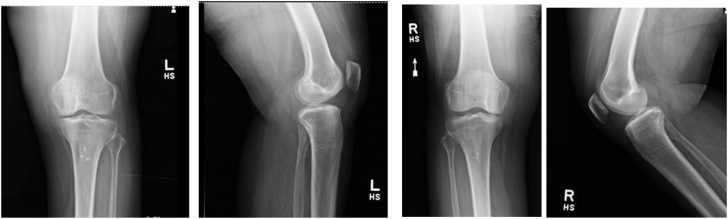
Weight-bearing films, 2017. Anteroposterior and lateral left and right knee radiographs were grossly normal.

**Figure 3:**
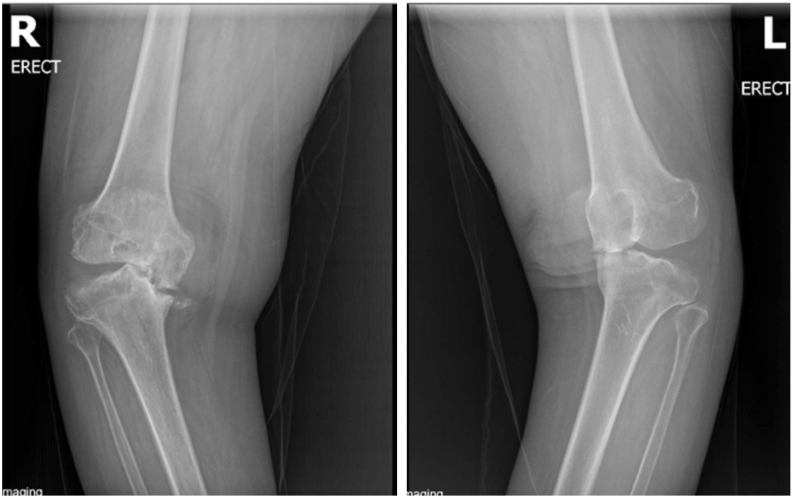
Weight-bearing films, 2020, showing severe varus collapse bilaterally. This is the time frame when the patient went from being able to ambulate with a walker to being wheelchair-bound due to pain and deformity.

Radiographs from 2020 were compared with those at the time of presentation to our orthopaedic clinic in 2025 (Fig. 4). The destructive changes had not progressed substantially, demonstrating relative radiographic stability of the bilateral knee arthropathy despite severe functional decline. This pattern of gradual decline and preserved bone stock is consistent with PPKA and contrasts with Charcot arthropathy, which is characterized by rapid and progressive joint destruction.

**Figure 4:**
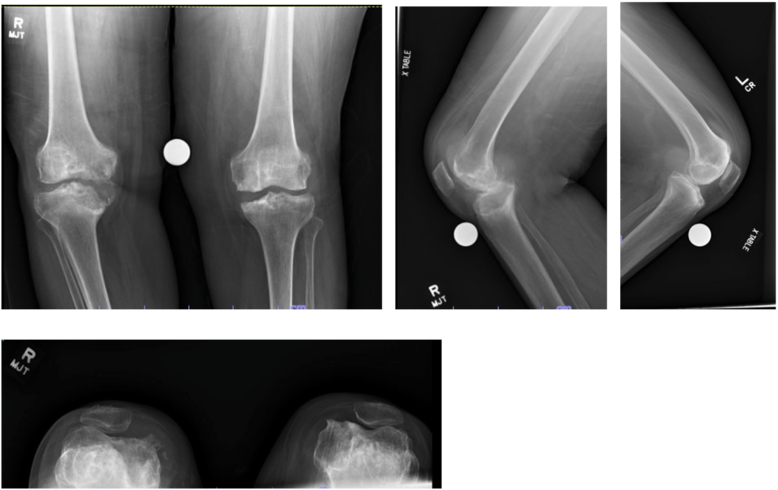
Non–weight-bearing radiographs obtained at initial presentation in 2025 demonstrating progressive osteoarthritic changes. The patient was unable to bear weight due to deformity. Medial tibial collapse is present in the right knee.

On physical examination, the patient was unable to stand, primarily related to profound instability of the knee joints bilaterally. She had diffuse global knee tenderness. Motor testing demonstrated 4-/5 in the left quadriceps, hamstrings, tibialis anterior, and gastrocnemius. She had 5-/5 strength in the right quadriceps, hamstrings, tibialis anterior, and gastrocnemius. The right knee had 50 degrees of varus collapse, which was passively correctable. The right knee hyperextended to 20 degrees with flexion of 110 degrees. The left range of motion was 50 degrees of hyperextension to 100 degrees of flexion. In both knees, her ligamentous exam was grossly unstable in the varus and valgus planes. The full extent of hyperextension and recurvatum instability was not assessed due to concerns of stress on the neurovascular structures. Preoperative radiographs confirmed severe varus malalignment bilaterally, and a noticeable medial tibial defect in the right knee. It is important to note in these radiographs that the bone stock of the knees is grossly preserved, as compared to what would be seen in Charcot arthropathy (Fig. 4).

She underwent staged bilateral H-TKA, with the right knee performed first and the left approximately 2.5 months later. Between surgeries, she was limited to slide-board transfers only. Figure 5 demonstrates the severity of the deformity intraoperatively. In both knees, the patient had severe instability and deformity necessitating high-constraint implants. There was thickened and hypertrophic synovium. The collateral ligaments in both knees were functionally incompetent. Long diaphyseal stems were used in both knees to bypass the compromised metaphyseal bone. Restoration of mechanical alignment and joint line was prioritized. Joint line errors are particularly detrimental in post-paralytic patients due to their typically weak quadriceps.

**Figure 5:**
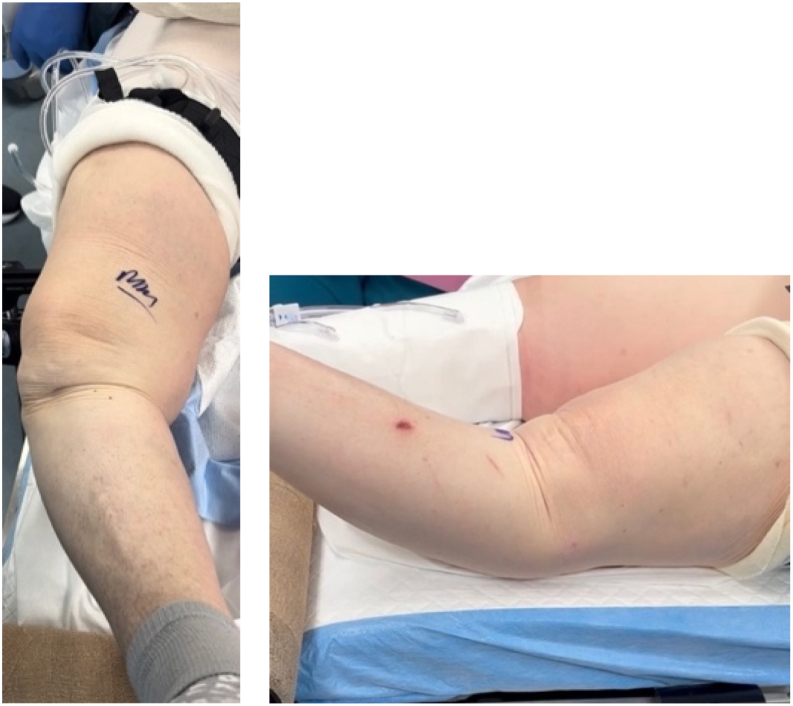
Intraoperative exam of the (a) right knee showing severe varus collapse (50 degrees) with moderate recurvatum deformity (20 degrees) and (b) the left knee showing severe recurvatum deformity (50 degrees).

The right knee tibial and femoral defects were Anderson Orthopaedic Research Institute stage 2B. Figure 6 demonstrates the medial tibial defect. The most technically challenging aspect of the procedure was the management of medial tibial plateau bone loss necessitating augmentation. A rotating H-TKA was used with cemented femoral and tibial components in addition to a 15 mm medial tibial augment with a tibial stem extension. The left knee tibial and femoral defects were AORI 2A. The implants consisted of a stemmed femoral component and a cemented tibial component with a metaphyseal cone. Intraoperative fluoroscopy confirmed appropriate alignment and patellar tracking for both knees.

**Figure 6:**
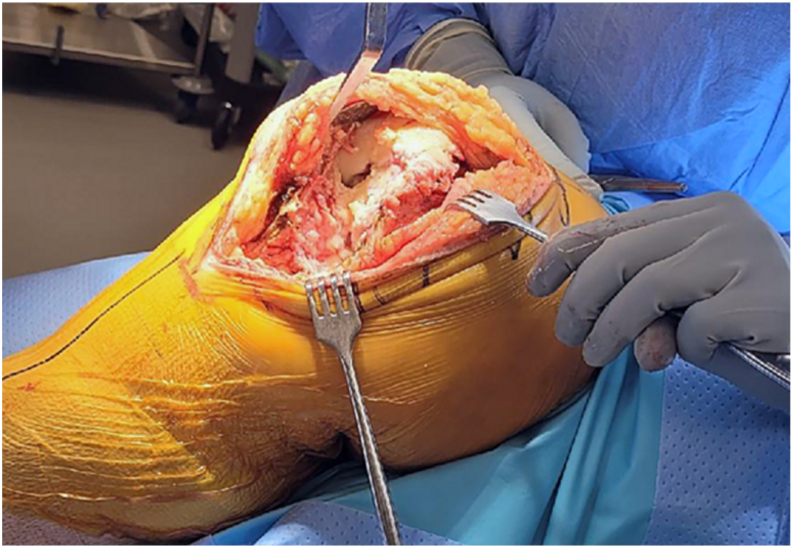
Intraoperative photo of the right knee demonstrating a defect in the medial tibial plateau.

Tibial fixation in both knees was achieved using 2 batches of high-viscosity bone cement, each mixed with 1g of vancomycin and 2g of cefepime. Similarly, 2 batches of high-viscosity bone cement were mixed with 1g of vancomycin and 2g of cefepime for femoral fixation. At the conclusion of the procedure, 2g of vancomycin and 4g of cefepime powder were placed in the wound. This antibiotic protocol was the treating surgeon’s preference for high-risk reconstruction. Figure 7 shows immediate postoperative films of both knees.

**Figure 7:**
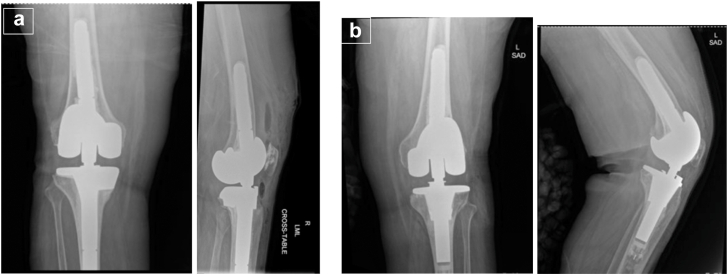
(a) Immediate postoperative images of the right H-TKA, and (b) Immediate postoperative images of the left H-TKA.

**Figure 8:**
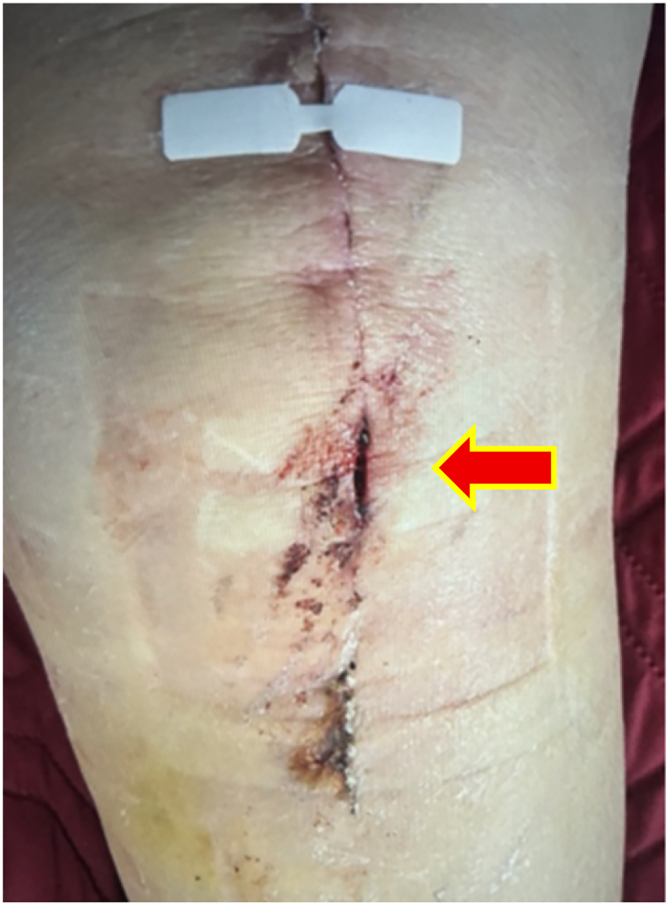
Superficial wound dehiscence at the mid portion of the surgical incision.

Three weeks after the left knee surgery, the patient developed mild swelling with a subcutaneous hemarthrosis. This led to an atraumatic superficial wound dehiscence at the midportion of the incision (Fig. 8). The patient’s inflammatory markers were within normal limits, and aspiration of the knee was not performed. She underwent exploration of the wound, and there was no deep fascial penetration. The wound was irrigated and closed. She was prescribed Keflex 500 mg four times daily for 1 week. Cultures were negative, and the incision healed uneventfully following reapproximation.

At 9 months following the right H-TKA and 7 months following the left H-TKA (Fig. 9), the patient demonstrated continued functional improvement and was ambulating with a walker for the first time since 2020. She remained actively engaged in physical therapy focused on gait training and strengthening.

**Figure 9:**
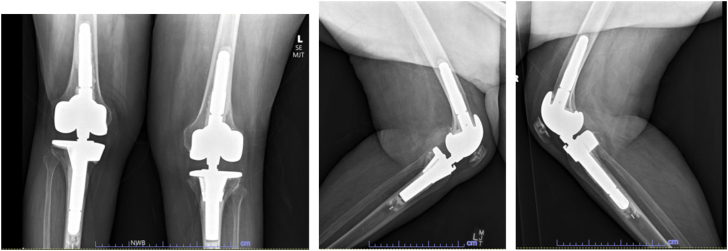
Last follow-up radiographs demonstrating the right knee at 9 months postoperative following hinged total knee arthroplasty (H-TKA) and the left knee at 7 months postoperative following H-TKA.

## Discussion

To our knowledge, this is the first reported case of bilateral PPKA that evolved over the course of 18 years, following a spinal cord ependymoma resection treated with staged H-TKA. Historically, a well-known cause of PPKA is post-poliomyelitis syndrome. [[1], [2], [3], [4], [5]] Spinal cord tumors represent a more modern and likely underrecognized cause of PPKA, as illustrated in this case. PPKA is driven by gradual chronic muscle imbalance leading to abnormal joint loading and resultant osteoarthritis. In contrast, Charcot arthropathy involves rapid joint destruction and fragmentation due to sensory loss and repetitive microtrauma. Charcot arthropathy can be caused by diabetes mellitus, neurosyphilis, syringomyelia, chronic alcoholism, spinal cord injury, cauda equina syndrome, severe spinal canal stenosis, and leprosy. [[6], [7], [8], [9], [10], [11], [12], [13], [14], [15]] Post-paralytic arthropathy of the spine has been described in patients with spinal cord injury or tumors; [[16], [17], [18]] however, post-paralytic arthropathy of peripheral joints, particularly the hip and knee, has been reported almost exclusively in association with poliomyelitis.

The surgical management of this case report itself is not unique, as the treatment strategy is consistent with what would be expected for the presenting deformity and instability. Rather, what makes this case unique is the underlying modern etiology of PPKA. PPKA outside the setting of post-polio syndrome has not been reported in the literature and may be partly due to underrecognition of this pathophysiology. There are currently no diagnostic criteria that exist for PPKA. The authors present in Table 1 a framework below of key identifying features of PPKA to consider when differentiating it from Charcot arthropathy. It is important to differentiate these 2 pathologies in order to guide patient expectations postoperatively. Additionally, it is essential to exclude primary inflammatory arthropathies (eg, rheumatoid arthritis, seronegative spondyloarthropathies, and chronic septic arthritis) as well as metabolic or deposition-related arthropathies (eg, hemochromatosis arthropathy, hemophilic arthropathy, and crystal deposition disorders) when establishing a diagnosis of PPKA.

**Table 1:** Identifying features to consider when differentiating PPKA from Charcot arthropathy.

Identifying feature	PPKA	Charcot arthropathy	Physical exam
Motor deficit	Present. Objective motor deficit in at least 1 major knee-controlling muscle group (MRC ≤4/5). Typically quadriceps weakness.	Not present. Normal quadriceps strength.	Focused MRC grading of quadriceps and knee stabilizers.
Sensation	Preserved.	Absent.	Great toe proprioception; Semmes-Weinstein monofilament testing.
Instability pattern	Functional recurvatum related to quadriceps weakness and compensatory mechanisms. Long-standing gait adaptations.	Uncontrolled, multiplanar instability. Typically varus and valgus; hyperextension may be present.	Careful gait observation.
Associated clinical findings	Muscle atrophy.	Warm, swollen joint.	Temperature comparison; assess for inflammatory signs.
Chronicity of deformity	Gradual progression over years. Bone stock generally preserved.	More rapid progression over months. Bone stock significantly reduced.	Serial imaging to assess progression rate.

MRC, Medical Research Council Scale for Muscle Strength.

Spinal pathology can cause PPKA or Charcot arthropathy, depending on whether motor or sensory pathways are affected. In PPKA, spine pathology can affect multiple regions of the spinal cord, including the anterior horn cells, motor roots, or corticospinal tracts. In this case, the patient’s descending corticospinal tracts were disrupted, which is in contrast to post-poliomyelitis syndrome where the anterior horn cells are targeted. Patients with PPKA present with a chronic course, with a history of lower extremity weakness affecting at least 1 muscle group crossing the knee for more than 1 year. Some other examples of causes of PPKA include spinal cord injury with residual motor weakness and post-laminectomy motor deficits. This contrasts with spinal pathology causing Charcot arthropathy, where patients lose protective sensation and proprioception; for example, syringomyelia expands from the central canal and disrupts crossing spinothalamic fibers, impairing pain and temperature pathways.

Figure 10 below illustrates the biomechanical compensatory mechanism of quadriceps weakness causing recurvatum. These changes can occur even with mild deficits of the quadriceps and not solely with profound weakness. During mid-stance, when the foot initially makes contact with the ground, the quadriceps counteract the flexion moment created when the ground reaction force passes posterior to the knee. When quadriceps strength is reduced, patients initially attempt to compensate by leaning the trunk forward. This shifts the center of mass anteriorly and moves the ground reaction force closer to, or slightly anterior to, the knee joint, reducing the external flexion moment. However, trunk flexion can only partially compensate for reduced quadriceps strength. Maintaining this posture requires increased activation of the hip extensors and is energetically inefficient. More importantly, trunk positioning alone cannot reliably control the knee throughout the entire stance phase. Even mild weakness, such as 4/5 quadriceps strength, can be insufficient for normal gait mechanics because stance requires continuous, precise control of the knee rather than intermittent maximal force production. Over time, patients adopt a more reliable compensatory strategy by allowing the ground reaction force to shift further anterior to the knee. This creates an external extension moment that drives the knee into hyperextension, allowing the limb to function as a stable column with minimal quadriceps activation. In this way, recurvatum becomes a passive stabilization strategy that replaces active quadriceps control.

**Figure 10:**
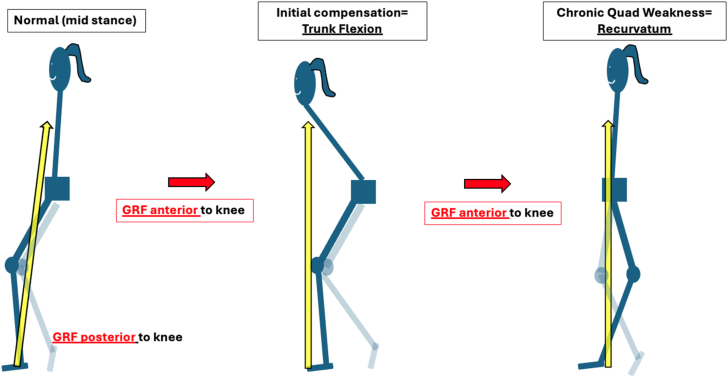
Illustrates the biomechanical compensatory mechanism by which quadriceps weakness leads to knee recurvatum. When quadriceps strength is insufficient to counteract the flexion moment created as the ground reaction force (GRF) passes posterior to the knee during stance, patients shift the ground reaction force anterior to the knee, creating an external extension moment that drives the knee into hyperextension and allows the limb to function as a stable column with minimal quadriceps activation.

Chronic hyperextension places repetitive stress on the posterior capsuloligamentous structures of the knee. Because the ground reaction force normally passes slightly medial to the knee during stance, this altered loading pattern also increases medial compartment stress. Over time, these forces contribute to progressive varus deformity and degenerative arthropathy, a pattern commonly observed in this case report in addition to post-poliomyelitis syndrome.

Historically, H-TKA has higher rates of complications such as periprosthetic joint infection and aseptic loosening when compared to less constrained knee arthroplasty options. [10,19] This is attributable to the implant transferring greater mechanical forces directly to the bone–implant interface [10,20], in addition to the technical complexity of these cases, which often involve multiply revised knees and prolonged operative times. In Charcot arthropathy specifically, there have been higher cases of periprosthetic joint infection reported in the setting of diabetes. [10,21]

In patients who have neuropathic joints, the senior author of this case report has only used H-TKA. This is in part due to lower constraint implants such as condylar constrained knee implants not being able to protect against worsening recurvatum. H-TKA is required for severe recurvatum deformities, which were seen in our case report. It is the perspective of the authors of this article that electromyography and nerve conduction study testing would not provide additional benefit or alter the ultimate treatment of this patient. The information gathered from a physical examination is sufficient to guide diagnosis and treatment.

Our patient’s bilateral severe ligamentous incompetence, substantial coronal and sagittal plane deformities, and chronic quadriceps weakness rendered conventional or semi-constrained implants inadequate to prevent recurrent instability. H-TKA allowed reliable restoration of stability and alignment while accommodating bone loss and minimizing reliance on compromised soft tissues. A rotating-hinge implant prevents varus/valgus collapse and pathologic recurvatum while providing a mechanical extension stop. In post-paralytic patients, restoring the joint line and mechanical axis is particularly critical given limited muscular compensation. The extension stop reduces the reliance on quadriceps strength. It is important to note that ambulation was achieved despite her persistent muscle weakness because of the implant design of a hinged prosthesis, providing a substitute for deficient soft tissues and neuromuscular control.

The right knee demonstrated AORI type 2B tibial bone loss with a contained but extensive medial metaphyseal defect and AORI type 2A femoral deficiency, which was managed using a medial tibial augment and diaphyseal stem fixation. On this side, a metaphyseal cone was not utilized. This decision was based on the size of the augment and the limited residual bone stock distal to the tibial augment, which was insufficient to accommodate a cone. In contrast, the left knee exhibited AORI type 2A tibial bone loss with compromised metaphyseal support, favoring the use of a porous tibial cone to restore structural stability and load transfer. The metaphyseal cone allowed for enhanced metaphyseal fixation, which allowed for a shorter stem. A metaphyseal cone was not utilized on the augmented side of the right knee due to inadequate metaphyseal geometry for cone accommodation following augmentation.

It is important to acknowledge that no preoperative or postoperative patient-reported outcome measures were collected, and follow-up is limited to 9 months. This represents a limitation of this case report, as outcome assessment relies primarily on the patient’s subjective report and findings from the most recent clinical examination rather than validated outcome instruments or longer-term follow-up data. The short follow-up period of 9 months limits our ability to assess long-term implant durability, mechanical survivorship, and late complications such as aseptic loosening or polyethylene wear, which are particularly relevant in constrained arthroplasty constructs. An important takeaway from this case report was to counsel the patient that surgery would not reverse the underlying neurologic condition and that she would still experience quadriceps weakness. The H-TKA implant will not replace motor control or restore the patient’s ability to actively extend the quadriceps. She continues to carry a risk of instability due to baseline motor weakness and ligamentous imbalance. She also has a higher infection risk due to altered soft tissue dynamics when compared to routine arthroplasty. She was counseled that, given her persistent quadriceps weakness, she will always be dependent on an assistive device (walker) for ambulation. We discussed the elevated risk of complications associated with constrained knee arthroplasty, including periprosthetic joint infection, aseptic loosening, and the potential need for revision surgery. Salvage procedures, including arthrodesis or amputation, were also discussed as possible outcomes in the event of severe complications.

In summary, this case represents the first reported instance of bilateral PPKA secondary to spinal cord tumor treated with staged H-TKA. Recognition of this distinct pathophysiology is critical to avoid misdiagnosis as Charcot arthropathy and to guide appropriate patient counseling and expectations. H-TKA provides a durable solution for managing severe deformity, instability, and bone loss in carefully selected post-paralytic patients.

### Current controversies and future considerations

• The long-term survivorship and complication profile of hinged implants in patients with PPKA without sensory loss are not well defined, highlighting the need for larger series and registry-level data.
• Standardized diagnostic criteria distinguishing post-paralytic arthropathy from Charcot arthropathy are lacking, and future studies should focus on defining imaging and clinical features.
• Optimal implant constraint in Charcot and PPKA is somewhat controversial in the literature, particularly regarding when to proceed directly to H-TKA vs attempting less constrained designs in the setting of neuromuscular imbalance. The senior author of this article favors H-TKA in post-paralytic and post-polio patients.

### Summary

• PPKA is a distinct and underrecognized cause of progressive osteoarthritis driven by muscle weakness and resulting abnormal joint mechanics.
• Severe ligamentous incompetence, coronal and sagittal plane deformities, and compromised neuromuscular control necessitate H-TKA to restore stability and alignment.
• Meticulous restoration of the joint line and mechanical axis, combined with high-constraint implants with extension control, can enable meaningful functional recovery and ambulation despite persistent muscle weakness, although many patients will continue to require lifelong ambulatory aids.

## Informed patient consent

The author(s) confirm that written informed consent has been obtained from the involved patient(s) or if appropriate from the parent, guardian, power of attorney of the involved patient(s); and, they have given approval for this information to be published in this case report (series).

## CRediT authorship contribution statement

**Jessica Nelson:** Writing – review & editing, Writing – original draft, Visualization, Validation, Methodology, Data curation, Conceptualization. **Austin Post:** Writing – review & editing, Writing – original draft, Visualization, Methodology, Investigation, Formal analysis, Conceptualization. **Michael Miller:** Writing – review & editing, Writing – original draft, Validation, Supervision, Conceptualization.

## Conflicts of interest

The authors declare there are no conflicts of interest.

For full disclosure statements refer to https://doi.org/10.1016/j.artd.2026.102058.

## Appendix A Supplementary data

Conflict of Interest Statement for Post

Conflict of Interest Statement for Nelson

Conflict of Interest Statement for Miller

## References

[1] Zari S., Sammouni O., Abdeljaouad N., Yacoubi H. (2023). Bilateral total knee replacement in a patient with poliomyelitis: a case report from Eastern Morocco. Cureus.

[2] Rahman J., Hanna S.A., Kayani B., Miles J., Pollock R.C., Skinner J.A. (2015). Custom rotating Hinge total knee arthroplasty in patients with poliomyelitis affected limbs. Int Orthop.

[3] Shah V.I., Patil J., Shetty G.M., Trivedi P. (2024). Primary total knee replacement using rotating Hinge implants in poliomyelitis: a case series and review of the literature. J Orthop Case Rep.

[4] Tardy N., Chambat P., Murphy C., Fayard J.M. (2014). Bilateral custom-fit total knee arthroplasty in a patient with poliomyelitis. Orthopedics.

[5] Tigani D., Calderoni E.F., Berti M., Comitini S., Amendola L., Pipino G. (2024). Long-Term results of third generation of rotating Hinge arthroplasty in patients with poliomyelitis. Prosthesis.

[6] Li G., Ding Y., Zhang C., Huang H. (2020). Syringomyelia with left knee charcot arthropathy: a case report. Br J Neurosurg.

[7] Kii S., Sonohata M., Mawatari M. (2023). Total knee arthroplasty for neuropathic arthropathy in a patient with leprosy. Mod Rheumatol Case Rep.

[8] Patel A., Saini A.K., Edmonds M.E., Kavarthapu V. (2018). Diabetic neuropathic arthropathy of the knee: two case reports and a review of the literature. Case Rep Orthop.

[9] Bae D.K., Song S.J., Yoon K.H., Noh J.H. (2009). Long-term outcome of total knee arthroplasty in Charcot joint: a 10- to 22-year follow-up. J Arthroplasty.

[10] Tibbo M.E., Chalmers B.P., Berry D.J., Pagnano M.W., Lewallen D.G., Abdel M.P. (2018). Primary total knee arthroplasty in patients with neuropathic (Charcot) arthropathy: contemporary results. J Arthroplasty.

[11] Zeng M., Xie J., Hu Y. (2016). Total knee arthroplasty in patients with Charcot joints. Knee Surg Sports Traumatol Arthrosc.

[12] Aydogdu S., Ozturk M., Sezak M., Sur H. (2012). Bilateral total knee arthroplasty with modified primary components in the management of neuropathic arthropathy related to chronic pancreatitis: a case with 5-year follow-up. Eur J Orthop Surg Traumatol.

[13] Tsamassiotis S., Windhagen H., Ettinger M. (2021). Bilateral primary total knee arthroplasty and reconstruction of the medial tibial Plateau by an asymmetric cone in a patient with charcot arthropathy. Case Rep Surg.

[14] Kopec K., Kusz D., Cielinski L., Wojciechowski P., Hajduk G. (2009). Bilateral neurogenic hip arthropathy. A case report. Neuro Endocrinol Lett.

[15] Karageorgos A., Papadopoulos A.X., Gelalis I.D., Matzaroglou C. (2020). Bilateral total knee arthroplasty for charcot arthropathy after Cauda Equine Syndrome: a case report. J Med Cases.

[16] Grassner L., Geuther M., Mach O., Bühren V., Vastmans J., Maier D. (2015). Charcot spinal arthropathy: an increasing long-term sequel after spinal cord injury with no straightforward management. Spinal Cord Ser Cases.

[17] C S., Dd C., P A. (1997). Charcot spine as a late complication of traumatic spinal cord injury. Arch Phys Med Rehabil.

[18] Pr F., D B., C O. (2022). Charcot arthropathy of the spine. J Am Acad Orthop Surg.

[19] Yoon J.R., Cheong J.Y., Im J.T., Park P.S., Park J.O., Shin Y.S. (2019). Rotating Hinge knee versus constrained condylar knee in revision total knee arthroplasty: a meta-analysis. PLoS ONE.

[20] Andreani L., Pianigiani S., Bori E., Lisanti M., Innocenti B. (2020). Analysis of biomechanical differences between condylar constrained knee and rotating hinged implants: a numerical study. J Arthroplasty.

[21] Parvizi J., Marrs J., Morrey B.F. (2003). Total knee arthroplasty for neuropathic (Charcot) joints. Clin Orthop.

